# Our 20-year experience with experimental colonic anastomotic healing

**Published:** 2018

**Authors:** Dimitrios Raptis, Manousos-Georgios Pramateftakis, Ioannis Kanellos

**Affiliations:** * 4th Surgical Department, Aristotle University of Thessaloniki, 54124, Thessaloniki, Greece

**Keywords:** colonic anastomoses, colorectal cancer, chemotherapeutic, anastomotic leakage

## Abstract

**Aim:** To present our experience with experimental colonic anastomoses and compare it with the results of other experienced researchers.

**Materials and Method:** The published experimental studies of our research group up to 1996, as well as results of other researchers in this field, are demonstrated and discussed. Different actions of administered substances on the anastomotic healing were compared and represented. Various chemotherapeutic agents were evaluated in experimental models without colorectal cancer as independent risk factors for the anastomotic healing. Moreover, numerous pharmaceutical agents such as steroids, immunomodulators, vasodilators and the use of fibrin glue are also assessed in detail.

**Results:** Cytostatics, as well as steroids, impair the colonic anastomotic healing, but the combined administration of other agents can reverse this negative effect. Fibrin glue seems to protect the colonic anastomosis, while iloprost could be a potential candidate for further exploration in patient trials. Tacrolimus, despite its immunosuppressive action, seems to promote the anastomotic healing. This observation could be useful for patients with inflammatory bowel disease under tacrolimus therapy, who undergo a non-elective colectomy. Obstructive conditions predispose to anastomotic insufficiency, and therefore, substances to avoid this threatening complication are also assessed. Tacrolimus and iloprost showed a remarkable action against anastomotic leakage under artificially obstructive conditions.

**Conclusion:** Further studies, especially in forms of clinical protocols, are necessary in order for these results to find their place in safe daily practice.

## Introduction

The anastomotic healing process is a complex multifactorial procedure that takes place in different stages and is primarily determined the net amount of collagen deposition at the anastomotic site, as well as the cross-linking of its fibers. Anastomotic insufficiency remains nowadays the most threatening and detrimental complication in traditional and laparoscopic colorectal surgery; it is associated with increased morbidity, mortality, reoperation, prolonged hospitalization and decreased quality of life, while early recognition is commonly based on non-objective criteria [**1**]. The overall anastomotic leak rate varies widely (1-24%). In experienced colorectal centers, leak rates were reported as 3.4% and 5.3% concerning colonic and rectal anastomoses, respectively [**2**][**3**]. In cancer patients, this is shown to affect negatively the long-term survival after potentially curative resections [**4**].

Various factors affect the anastomotic healing, and they are divided into systemic and local ones. Systemic factors include age, nutritional status, comorbidity and chemoradiation, whereas local factors include impaired blood supply at the anastomotic site and surgical technique. Of course, colorectal cancer, as a systemic disease, negatively affects the anastomotic integrity in a multifactorial, well-known way.

Our research group started to publish its experience in experimental anastomotic healing in 1996. Since 2012, we are assessing the impact of numerous factors on the anastomotic progress under obstructive conditions, as it is well-documented that anastomotic leak rates are dramatically increased under the above-mentioned conditions, especially after emergency colectomies. Our research group focused mainly on agents used in all-day practice, such as cytostatics, immunosuppressors, vasodilators and growth factors. In this way, these findings could be easily translated into clinical cases in humans.

We did not evaluate the effect of chemotherapeutic agents in models with colorectal malignancy, as our tendency was to assess these as independent risk factors for the anastomotic healing progress.

We aim to demonstrate a collective summary of our experience throughout the last decades.

## Experimental experience of our research group Methods

All experimental studies were approved by the Ethical Committee of the Department of Veterinary Services of the Prefecture of Thessaloniki and the Veterinary School of the Aristotle University of Thessaloniki. We used Wistar-albino rats, obtained from the same breeding center for each study and all of the animals received humane care in accordance with the “Principles of Laboratory Animal Care”, formulated by the National Society for Medical Research and the “Guide for the Care and the Use of Laboratory Animals” prepared by the Institute of Laboratory Animals Resources and published by the National Institute of Health.

An end-to-end anastomosis using 6/0 polypropylene interrupted sutures in an extramucosal single-layer fashion was always performed. The abdominal wall was closed in one-layer using 3/0 silk sutures. Depending on the study, the animals received the substances either intraperitoneally or subcutaneously.

The macroscopic examination included the anastomotic integrity, the presence of perianastomotic abscesses, the formation of adhesions and the existence of peritonitis. The results were evaluated blindly according to the scale of van der Hamm et al. [**5**]. At the microscopic level, inflammatory cell infiltration, fibroblast activity, neoangiogenesis and collagen deposition were assessed in a blind fashion using the Ehrlich and Hunt numerical scale, as modified by Philips et al. [**6**]. The biochemical estimations included the hydroxyproline concentration and collagen type I activity, to assess in further details the fine balance between collagen formation and degradation.

Bursting pressure is the most reliable factor for the evaluation of the anastomotic strength, and it was evaluated in every study, immediately after the sacrifice of the animals, using a method that has been described elsewhere [**7**]. Moreover, the location of the anastomotic leakage or rupture during bursting pressure measurement was recorded.

## Substances that impair the anastomotic strength.

5-Fluorouracil (5-FU): In 1996, Kanellos et al. assessed the effect of intraperitoneal administration of 5-FU alone or in combination with folinic acid, intraoperatively and daily for the first two postoperative days. The anastomotic integrity was negatively affected, and adhesion formation, inflammatory reaction and perianastomotic abscess rates were significantly increased. Moreover, collagen formation and fibroblast activity were impaired. The coadministration of folinic acid (leucovorin) did not aggravate this negative effect [**8**]. In a further study published in 1997, the combined administration of 5-FU with interferon-alpha led to similar results [**9**]. On the other hand, delayed intraperitoneal administration of 5-FU combined with folinic acid or interferon, starting on the fourth postoperative day, had no adverse effects on the healing process [**10**][**11**]. 

*Irinotecan:* The effects of intraoperative intraperitoneal administration of irinotecan were assessed by Pramateftakis et al. [**12**]. On the eighth postoperative day, the anastomotic bursting pressures, the neoangiogenesis, as well as the hydroxyproline levels, were significantly decreased. The irinotecan-treated animals suffered extensive adhesion formation, while the inflammation was significantly increased. In a similar experimental model, the combined administration of irinotecan and 5-FU intraoperatively was also evaluated by our research group and shown that on the eighth postoperative day the negative effect of the combination was significantly increased compared to the single use of each substance [**13**].

*Oxaliplatin:* For the evaluation of the influence of oxaliplatin on colonic anastomotic healing, a similar rat model was used. Oxaliplatin was administered intraperitoneally after the anastomosis was performed as well as once a day for seven days, followed by the animals’ sacrifice on the eighth postoperative day. Anastomotic dehiscence was noted only in the oxaliplatin group, but the difference was not statistically significant compared to the control group. Anastomotic bursting pressures, neoangiogenesis, fibroblast activity, collagen deposition, hydroxyproline concentration and -interestingly- the inflammatory reaction were significantly decreased [**14**]. After the intraoperative administration of oxaliplatin in only one dose, the results were similar, but the inflammatory infiltration was significantly increased on the eighth postoperative day, as it was demonstrated by Kanellos et al. [**15**]. With a similar study protocol, the combination of oxaliplatin and 5-FU was demonstrated to further impair the anastomotic healing progress. Bursting pressures, hydroxyproline concentration, collagen amount and fibroblast activity after combined administration were significantly lower. Moreover, the adhesion formation score and leucocytosis were increased [**16**].

*Hydrocortisone:* In a rat model, Mantzoros et al. evaluated the impact of hydrocortisone on the colonic anastomotic healing. Hydrocortisone was injected intraperitoneally seven days preoperatively, as well as seven days after the anastomosis was performed. The bursting pressures, as well as the evaluated histological parameters such as hydroxyproline concentration, collagen deposition and inflammatory cell infiltration, were significantly reduced. Moreover, a 25% increase in the anastomotic leakage rate in the cortisone-treated animals was noted. These data clearly demonstrate that the perioperative administration of steroids adversely affects the anastomotic healing progress [**17**] (**Table 1**).

**Table 1:** Substances impairing the anastomotic strength

Substance	Year of the study	Way and day of administration	Number of male Wistar rats/ day of sacrifice	Bursting pressure (significance with p-value)
Chemotherapeutic agents				
[8]5-FU (20mg/kg)	1996	Intraperitoneally 0*-2	63/ 3, 5, 8	↓ p<0.05 (compared with the control-group)
[8]5-FU (20mg/kg)+ FA (2mg/kg)	1996	Intraperitoneally 0*-2	63/ 3, 5, 8	↓ p<0.05 (compared with the control-group)
[9] 5-FU (20mg/kg)+ interferon-a (45,000IU/kg)	1997	Intraperitoneally 0*-2	57/ 3, 5, 8	↓ p<0.05 (compared with the control-group)
[10] 5-FU (20mg/kg)+; FA (2mg/kg)	1998	Intraperitoneally 0*-3 or 4-7	70/ 8	↓ p<0.05 (compared with the control-group, early administration); p>0.05 (compared with the control-group, delayed administration)
[11] 5-FU (20mg/kg)+; Interferon-a (45,000IU/kg)	1998	Intraperitoneally 0*-3 or 4-7	70/ 8	↓ p<0.05 (compared with the control-group, early administration); p>0.05 (compared with the control-group, delayed administration)
[12] Irinotecan (3mg/kg)	2007	Intraperitoneally 0*	30/8	↓ p<0.001 (compared with the control-group)
[13] 5-FU (20mg/kg)+ Irinotecan (3mg/kg)	2011	Intraperitoneally 0*-1	60/8	↓ p<0.001 (compared with the control, the irinotecan, as well as with the 5-FU-group)
[14]Oxaliplatin (2.4mg/kg)	2010	Intraperitoneally 0*-7	30/10	↓ p<0.001 (compared with the control-group)
[15]Oxaliplatin (2.4mg/kg)	2008	Intraperitoneally 0*	30/8	↓ p<0.001 (compared with the control-group)
[16] 5-FU (20mg/kg)+; Oxaliplatin (2.4mg/kg)+	2011	Intraperitoneally 0*-1	60/8	↓ p<0.001 (compared with the control, the oxaliplatin, as well as with the 5-FU-group)
Non-chemotherapeutic agents				
[17] Hydrocortisone (5mg/kg)		Intraperitoneally 7 days preoperatively and 0*-7 postoperatively	40/8	↓ p<0.001 (compared with the control-group)

* day 0 means intraoperative; 5-FU: 5-fluorouracil, FA: folinic acid (leucovorin). Significance level: p<0.05

## Substances that enhance the anastomotic strength.

*Fibrin glue:* In 2003 Kanellos et al. evaluated the application of fibrin glue immediately after the colonic anastomosis was performed. All of the animals were sacrificed on the eighth postoperative day, and the bursting pressure mean rates were significantly increased in fibrin-coated anastomoses. Interestingly, the anastomotic leak rates were not significantly different compared to the control ones [**18**]. In another study, our research group investigated whether the covering of the anastomosis with fibrin glue could reverse the adverse effect of the intraperitoneal administration of 5-FU. All rats were sacrificed on the eighth postoperative day. The leakage rates were significantly decreased in the fibrin glue-coated anastomoses; the bursting pressures, formation of new vessels and fibroblast activity were also positively affected by the application of fibrin glue. Moreover, the fibrin glue presence reduced the intra-abdominal adhesion formation score. The authors concluded that the application of fibrin glue could significantly reverse the impairment of anastomotic healing after early administration of 5-FU as monotherapy [**19**]. Similar results were demonstrated after the combined administration of 5-FU and leucovorin in the early postoperative phase when the anastomoses were covered with fibrin glue. No leakages were noted, while the bursting pressures were significantly higher in comparison with the control group. Neoangiogenesis, as well as the fibroblast activity, were enhanced, whereas the mean adhesion formation score was significantly lower, demonstrating that the application of fibrin glue could prevent the deleterious effect of early chemotherapy [**20**]. As mentioned previously, our research group demonstrated that the additional intraperitoneal injection of interferon does not aggravate the adverse effect of postoperative intraperitoneal administration of 5-FU in the early postoperative phase [9]. The impact of fibrin glue after the injection of this chemotherapeutic combination was also assessed. In 2007, we demonstrated that the application of fibrin glue could lead to increased bursting pressures and neoangiogenesis, whereas inflammatory cell infiltration, collagen deposition, and fibroblast activity were not significantly affected. Moreover, in the fibrin glue-treated animals, no leakage incidence was noted [**21**]. In another study conducted in our laboratory, it was demonstrated that the application of 2-octyl cyanoacrylate (a type of adhesive glue) could provide a safe sutureless anastomosis, equal to the conventional one, as it resulted from our data (under experimental conditions) [**22**].

*Insulin-like growth factor I (IGF-I):* In an experimental study, Zacharakis et al. investigated the contribution of insulin-like growth factor I in the colonic healing process, when injected intraperitoneally on day 2, 4 and 6 postoperatively after colonic resection. Animals were sacrificed on the eighth postoperative day. The bursting pressures, hydroxyproline concentration and fibroblast activity were significantly higher after the administration of IGF-I, concluding that the intraperitoneal administration of IGF-I stimulates the healing of colonic anastomoses [**23**]. In a further study of the same group of researchers, demonstrated that the intraperitoneal injection of IGF-I on day 2, 4 and 6 postoperatively after early administration of 5-FU mediates the adverse effects of chemotherapeutic agents partially, leading to increased bursting pressures, as well as hydroxyproline tissue concentrations [**24**].

*Iloprost:* In a further experiment, we assessed the effects of iloprost, a stable prostacyclin analog. Iloprost enhanced the anastomotic integrity in the early healing phase. As expected, bursting pressures were increased only in the early period of the anastomotic healing, namely on the fourth or fifth postoperative day, depending on the study. Neoangiogenesis was also significantly promoted at the anastomotic site [**25**][**26**]. The major question that appeared was whether iloprost could also counteract the negative effect of intraperitoneally administrated chemotherapeutics in the early postoperative period. In our experimental model, when injected intraperitoneally, iloprost was able to reverse the negative effects of combined 5-FU and leucovorin, increasing bursting pressures, neoangiogenesis, hydroxyproline levels and collagen deposition at the anastomotic site. Moreover, significantly decreased leakage rates were noted in the iloprost-treated animals [**27**]. The effects of this angiodilator have also been investigated under obstructive conditions. Obstructive ileus is a major factor causing local and systemic alterations and subsequently leading to impaired anastomotic healing. In a recent study by Galanopoulos et al., it was shown that iloprost could reverse the adverse effects of obstructive ileus on the anastomotic healing. Specifically, bursting pressures, neoangiogenesis, fibroblast activity and hydroxyproline concentration were significantly increased on the fourth as well on the eighth postoperative day. We demonstrated that iloprost promotes not only the angiogenic activity but also collagen formation and deposition, indicating enhanced healing. [**28**].

*Tacrolimus:* Since tacrolimus, a relatively new immunosuppressant agent, was approved for patients suffering from inflammatory bowel disease (IBD), its effect on colonic anastomoses was also evaluated. In a recently published study, Raptis et al. (in an experimental model) subcutaneously administered tacrolimus intraoperatively and daily for 3 or 7 days, depending on the study group. On the fourth postoperative day, bursting pressures, fibroblast activity, angiogenesis and hydroxyproline concentration were significantly higher in the tacrolimus-treated animals. Additionally, on the eighth day, the inflammatory cell infiltration and the collagenase I concentrations were significantly decreased, concluding that tacrolimus impairs not only the inflammatory response but also collagen degradation [**29**]. Under obstructive conditions, on both days of the study, the bursting pressure, fibroblast activity and collagen deposition were significantly higher in tacrolimus versus control groups. Moreover, the inflammatory cell infiltration was significantly decreased. Collagenase I concentrations were significantly lower only on the fourth day, while hydroxyproline concentrations were significantly reduced only on the eighth day. We concluded that tacrolimus impairs the inflammatory response in the early phase of healing and impairs collagen degradation; at a later stage, it promotes collagen formation and, subsequently, reconstruction of the intestinal wall [**30**] (**Table 2**).

**Table 2:** Substances enhancing the anastomotic strength

Substance	Year of the study	Way and day of administration	Number of male Wistar rats/ day of sacrifice	Bursting pressure (significance with p-value)
[18]Fibrin glue	2003	Around the anastomosis	36/8	p<0.001 (compared with the control-group)
[19]Fibrin glue + 5-FU (20mg/kg)	2004	Intraperitoneally 0*	64/8	p<0.001 (compared with the control-group, as well as with the 5-FU group)
[20]Fibrin glue + 5-FU (20mg/kg) + LEV (2mg/kg)	2006	Intraperitoneally 0*-3	60/8	p<0.001 (compared with the control group, as well as with the 5-FU + LEV group)
[21]Fibrin glue + 5-FU (20mg/kg) + IFN (45,000IU/kg)	2007	Intraperitoneally 0*-7	60/8	p<0.001 (compared with the control group, as well as with the 5-FU + LEV group)
[22]Sutureless anastomosis covered with 2-octyl cyanoacrylate	2002	Around the anastomosis	40/7	P= 0,897 (sutureless covered with 2-octyl cyanoacrylate compared with suture anastomosis)
[23]IGF-I (2mg/kg)	2007	Intraperitoneally 0*, 2, 4 and 6	40/7	p<0.001 (compared with the control group)
[24]IGF-I (2mg/kg) + ; 5-FU(20mg/kg)	2008	Intraperitoneally 0*, 2, 4 and 6	80/7	P=1.000 (compared with the control group); p<0.001 (compared with the 5-FU group)
[25]Iloprost(2mg/kg)	2007	Intraperitoneally 0*-4 or; 0*-7	40/5 or 8	p<0.001 (compared with the control group on the 5th postoperative day); p=0.165 (compared with the control group on the 8th postoperative day)
[26]Iloprost(2μg/kg)	2011	Intraperitoneally 0*-3 or; 0*-7	40/4 or 8	p<0.001 (compared with the control group on the 4th postoperative day); p=0.147 (compared with the control group on the 8th postoperative day)
[27]Iloprost(2μg/kg)+ 5-FU (20mg/kg) + LEV (2mg/kg)	2007	Intraperitoneally 0*-4 or; 0*-7	80/5 or 8	p<0.001 (compared with the 5-FU + LEV group on the 5th postoperative day); p=0.126 (compared with the 5-FU + LEV group on the 8th postoperative day);
[28]Iloprost(2μg/kg) + ileus	2014	Intraperitoneally 0*-3 or; 0*-7	80/4 or 8	p=0.001 (compared with the ileus group on the 4th postoperative day); p<0.001 (compared with the ileus group on the 8th postoperative day)
[29]Tacrolimus (0.1 mg/kg)	2012	Subcutaneously 0*-3 or; 0*-7	40/ 4 or 8	p<0.001 (compared with the control group on the 4th postoperative day, as well on the 8th postoperative day);
[30]Tacrolimus (0.1 mg/kg) + ileus	2012	Subcutaneously 0*-3 or; 0*-7	40/ 4 or 8	p=0.001 (compared with the ileus group on the 4th postoperative day); p<0.001 (compared with the ileus group on the 8th postoperative day)

*day 0 means intraoperative; 5-FU: 5-fluorouracil, LEV: leucovorin (folinic acid), IFN: interferon, IGF-I: Insulin-like growth factor I; Significance level: p<0.05

## Discussion

Nowadays, anastomotic leakage still remains a much-feared complication after colonic resection and decreases the cancer-related survival [**31**]. Since 1996, our research group has extensive experience concerning the experimental anastomotic assessment, under various conditions

Chemotherapeutic agents are well-known to affect the anastomotic healing negatively after intraoperative administration. Cytoreductive surgery (CRS) with hyperthermic intraperitoneal chemotherapy (HIPEC) is a safe and efficient additive treatment, performed only in specialized centers in indicated cases of colorectal peritoneal carcinomatosis [**32**][**33**][**34**][**35**][**36**]. The intraperitoneal application of chemotherapeutic agents adversely affects the progression of anastomotic healing, and leak rates ranging from 10.4% to 20% are reported [**37**][**38**].

Thus, we evaluated the effects of intraperitoneally administrated chemotherapy, as an independent risk factor in rat models, without colorectal cancer. In our study, where 5-FU was administrated immediately after the formation of the anastomosis, and daily for the first two postoperative days, the anastomotic integrity was adversely affected. Moreover, the combination with folinic acid (leucovorin) or interferon alpha led to similar results. In contrast to this observation, Haciyanli et al. demonstrated that leucovorin does not affect to a greater extent the anastomotic healing, after intravenous or intraperitoneal administration combined with 5-FU [**39**]. The most recent study was published by Ozel et al. and they concluded that the preoperative intravenous administration of 5-FU negatively affects the anastomotic healing, while the intraperitoneal administration does not affect the healing progress [**40**]. In two further studies, we demonstrated that delayed intraperitoneal administration of 5-FU plus leucovorin or interferon, has no adverse effects on anastomotic healing, confirming the theory that the colonic anastomosis lies at its lowest mechanical and physiological “strength” levels on the third postoperative day and gradually increases, reaching almost normal values on the seventh postoperative day.

The application of fibrin glue at the anastomotic site, on the other hand, reverses the adverse effect of 5-FU on the eighth postoperative day. Similar results were demonstrated after co-administration of 5-FU and IGF-I. Uludag et al. administrated 5-FU and had anastomoses covered by amniotic membranes. They concluded that covering colon anastomoses with amniotic membrane prevents leakage and reverses the negative effects of 5-FU [**41**]. In an experimental model in rabbits, Tümer et al. administrated 5-FU and 5-FU plus zinc for four days postoperatively. Zinc reversed the adverse effects of 5-FU [**42**]. Additionally, Colak et al. concluded that concomitant octreotide might prevent the side effects of 5-FU, such as diarrhea, adhesions and delayed anastomotic healing [**43**].

Irinotecan is recommended for advanced metastatic colorectal cancer as initial therapy in patients suited for intensive therapy [**44**]. We assessed the effects of Irinotecan as monotherapy or in combination with intraperitoneal 5-FU in a rat model. Irinotecan significantly decreased anastomotic bursting pressures, as well as neoangiogenesis, while adhesion formation and inflammatory infiltration were increased. The combination of Irinotecan and 5-FU negatively affected the healing progress, reducing the bursting pressures, while most of the microscopic assessments were further affected. In an experimental study that took place in 2008, Eyol et al. demonstrated in a rat model that Irinotecan could be safer than doxorubicin for the chemoembolization of colorectal liver metastases [**45**].

Oxaliplatin is recommended as adjuvant chemotherapy for high-risk stage II and stage III disease in combination with leucovorin and 5-FU (FOLFOX) [**42**]. It forms both inter- and intra-strand cross-links in DNA, which prevent replication and transcription. In an experimental rat model, Hribaschek et al. showed that oxaliplatin inhibited tumor growth after intraoperative and early postoperative application but only by intraperitoneal application [**46**]. In our first study, oxaliplatin was administrated in a single dose immediately after the anastomosis was formed and seemed to impair the anastomotic healing significantly. In order to evaluate this observation, with a similar research model, we administrated oxaliplatin immediately intraoperatively, as well as daily until the day of sacrifice. We further observed that after continuous postoperative administration, the inflammatory reaction was decreased, while, as expected, the anastomotic integrity was impaired. Ersoy et al. compared the impact of oxaliplatin and 5-FU on the healing of colonic anastomoses. 5-FU was administrated on day 1 and 3 and oxaliplatin on day 1 and 5. Oxaliplatindid not significantly affect the bursting pressures, but hydroxyproline concentrations in rats treated on day 1 were significantly lower than those treated with the agent on day 5, suggesting that a delayed administration could eliminate the adverse effects at the anastomotic site [**47**].

Steroid therapy is well-known to interfere with wound healing progress. Patients who are suffering from inflammatory bowel disease usually get treated with steroids. Thus, we injected hydrocortisone intraperitoneally (5mg/kg body weight) on a daily basis for 7 days pre- and 7 days post-operatively and we have shown that the administration of hydrocortisone affects the anastomotic healing negatively. In 1991, Mastboom et al. concluded that colonic anastomosis healing remains unaffected after short-term administration of methylprednisolone at a dose of 2.5 or 10mg/kg body weight [**48**]. Eubanks et al. studied the effect of equipotent doses of dexamethasone, hydrocortisone, and methylprednisolone on anastomotic healing. They concluded that dexamethasone slows the rate of wound healing, but short-term high-dose steroid therapy does not decrease the anastomotic integrity. Moreover, bursting pressures of the intact cecum were lower in all steroid-treated animals [**49**]. In a recent study, Letowska et al. used morphometric and fractal parameters to assess the effects of dexamethasone, 5-FU and interferon on colonic anastomotic healing and concluded that dexamethasone-induced morphometric and macroscopic alterations were considered the most detrimental [**50**]. In another study from Turkey, the authors concluded that low and high dose in short- or long-term administration produced adverse effects on the anastomotic healing, but the most prominent negative effect was associated with high-dose, long-term administration [**51**].

Fibrin glue, also called fibrin sealant, has not been consistently successful to prevent anastomotic leakage in the absence of a chemotherapeutic. Van der Vijver et al. concluded that there is no justification for using fibrin glue on patent anastomoses constructed under low-risk conditions [**52**]. In a study from Mexico, the authors investigated the effects of different concentrations of fibrinogen and thrombin on bursting pressure, leaks, dehiscence, and morphology of high-risk ischemic colonic anastomoses using fibrin glue. They concluded that fibrin glue with low fibrinogen content normalizes the bursting pressure of high-risk ischemic left colonic anastomoses in rats on the fifth postoperative day [**53**]. We evaluated the application of fibrin glue immediately after the anastomoses were formed and we concluded that bursting pressures were increased, but the anastomotic leak rates were not significantly affected. Under the presence of 5-FU, in a study from our research group, fibrin reversed the adverse effects of 5-FU at the anastomotic site. Similar results were demonstrated after the administration of 5-FU and leucovorin, in the early healing phase, when the anastomoses were coated with fibrin glue. Fibrin glue was also applied in combination with interferon (INF) and 5-FU; it managed to promote the anastomotic healing process.

Insulin-growth factor I belongs to the IGF family and we assessed the hypothesis that IGF-I could also enhance the anastomotic healing of the colon. We demonstrated that the intraperitoneal injection of the substance on days 2, 4 and 6 after colonic resections resulted in increased anastomotic integrity. Petersen et al., on the other hand, concluded that IGF-I increases the postoperative body weight and stimulates collagen deposition in left colonic anastomoses, whereas the anastomotic strength may be unaffected by IGF-I treatment [**54**]. Combined administration of IGF-I and 5-FU, in the same model as ours, partially mediated the adverse effect of the cytostatic agent. In an interesting study by Rijken et al., IGF-coated sutures were shown to improve important aspects of the anastomotic healing in rats [**55**].

Iloprost is a stable analog of prostacyclin, a potent dilator and an inhibitor of platelet aggregation. It has been shown to attenuate leucocyte adherence in intestinal venules and improve the microvascular blood supply. We hypothesized that iloprost administration could accelerate the anastomotic healing, ensuring an adequate blood-supply to the anastomotic site. In two experimental studies, we demonstrated the positive effect of iloprost on colonic anastomotic healing. Iloprost improved the mechanical strength of the anastomoses in the early postoperative phase, resulting in increased bursting pressures on the third as well as on the fourth postoperative day. Due to the fact that neoangiogenesis was significantly promoted, we concluded that iloprost enhances the anastomotic healing acting as a vasodilator, ensuring adequate blood supply to the anastomotic site.

Bostanoglou et al. concluded that iloprost, administered in combination with 5-FU could counteract and reverse the adverse effect of cytostatics. They showed that iloprost significantly improved the anastomotic strength only at the histopathological level, while no significant difference in bursting pressures was noted [**56**]. Both studies agree that iloprost promotes the blood supply in the early healing stage. In a further study of our group, we investigated whether iloprost could also successfully promote the anastomotic healing after combined administration with 5-FU and leucovorin. The application of iloprost increased the bursting pressures and all histological parameters of the healing process on the fifth as well as on the eighth day of study. Moreover, iloprost successfully interacted and partially reversed the negative effect of chemotherapeutics.

Tacrolimus, widely known as FK-506, is an immunosuppressant macrolide, used in organ transplantations. It inhibits interleukin-2 (IL-2) gene expression and causes effects at the cellular level, such as degranulation and apoptosis in leukocytes. Furthermore, it impairs the transcription of pro-inflammatory cytokines, acting as an anti-inflammatory factor and immunomodulator. Besides, tacrolimus shows an inhibitory action in nitric-oxide production, especially in colonic epithelial cells and induces ischemia-reperfusion injury. Numerous studies showed that tacrolimus promotes the release of various growth factors. Concerning the fact that tacrolimus is also approved as therapy in inflammatory bowel diseases, the effects of this immunomodulator on the colonic anastomotic healing were also to be evaluated in further details. Kiyama et al. demonstrated that tacrolimus significantly increased colonic bursting pressures in a dose-independent way. Collagen activity was increased, as it was shown microscopically; on the other hand, interestingly, collagen deposition was reduced [**57**]. Schäffer et al. investigated the effects of tacrolimus on dermal and intestinal wound healing and concluded that tacrolimus impaired dermal healing by decreasing the expression of TGFβand increasing the expression of IFN-γ and TNF-α. In contrast, colonic anastomoses were not impaired, indicating that tacrolimus differentially affects tissue healing and expression of cellular mediators in dermal and intestinal wound [**58**]. In contrast to these results, Martine et al. concluded that tacrolimus affects neither the abdominal wall wound healing nor intestinal anastomoses [**59**]. On the fourth postoperative day of our study, bursting pressures, fibroblast activity, neoangiogenesis and hydroxyproline concentration were significantly increased after subcutaneous administration of tacrolimus, whereas inflammatory reaction and collagenase I concentration decreased significantly. On the eighth postoperative day, bursting pressures and hydroxyproline concentration increased significantly, whereas inflammatory infiltration and collagenase I concentration were significantly lower. The collagen deposition, exactly as Kiyama et al. showed, was not affected. Our results suggest that tacrolimus promotes the healing of colonic anastomosis and impairs not only the inflammatory response but also collagen degradation.

With regard to iloprost and tacrolimus as anastomotic promoters, a meta-analysis by Oines et al. showed that iloprost increased the weighted mean of the early bursting pressure of colonic anastomosis in male rats by 60mm Hg and tacrolimus by 29mm Hg, respectively. They suggest that despite the fact that both the agents enhance the anastomotic integrity, only iloprost could be a potential candidate for further exploration, as tacrolimus also acts as an immunomodulator [**60**]. In our opinion, tacrolimus in a low dose shows a completely reversible action and the topical application is also to be evaluated, as this immunomodulator is absorbable from the colonic mucosa.

According to retrospective studies, 10-25% of patients admitted with acute colonic obstruction require urgent intervention. In approximately 60% of these patients, colorectal cancer is the common cause. In experienced colorectal centers, a one-step and oncologically adequate -according to the latest guidelines- procedure should be ideally undertaken, in case curative surgery is elected. An emergency colectomy is associated with increased morbidity and mortality rates, and obstructive ileus is well-documented to impair the anastomotic strength, due to the upregulation of metalloproteinases and reduced collagen synthesis. Moreover, systemic and local alterations induced under obstructive conditions disturb the blood supply at the anastomotic site [**61**][**62**][**63**]. In two recently published studies, we investigated whether iloprost and tacrolimus could also act as promoters under obstructive conditions, with the same experimental model mentioned before.

Under artificially obstructive conditions, iloprost significantly increased the mean rates of bursting pressure, hydroxyproline concentration and neoangiogenesis on the fourth as well as on the eighth postoperative day. Moreover, the collagen amount was significantly increased on the eighth day, suggesting that iloprost promotes not only the angiogenic activity but also the formation of collagen in case of obstruction.

Tacrolimus has similarly enhanced the anastomotic healing, resulting in increased bursting pressures in both days of study. It promoted collagen formation and reduced the inflammatory cell infiltration on the fourth as well as on the eighth day of study. Interestingly, collagenase I activity was reduced only on the fourth day, while the tissue concentration in hydroxyproline was increased only on the eighth day, suggesting that in the early phase of healing, tacrolimus impairs the inflammatory response while later it enhances collagen formation and promotes the bowel-wall reconstruction.

## Conclusion

Several substances were evaluated and shown to affect the colonic anastomosis in a multifactorial way. It is already well-documented that cytostatic agents impair the healing process and further effort, eventually in the form of clinical trials, is necessary in order to establish modern combinations of agents resulting in an adequate oncological result and reduced morbidity. The impact of the novel chemotherapeutic agents, e.g., antiangiogenic factors and furthermore of the modern pharmaceutical combinations for the treatment of inflammatory diseases on the anastomotic healing should also be assessed in detail.

## Conflict of interest

The authors declare no conflict of interest.
